# The development of cardiology in South Africa

**Published:** 2009-02

**Authors:** Andries Brink

**Affiliations:** Editor-in-Chief (South Africa)

The discipline of cardiology in South Africa has advanced rapidly over the last 70 years. This editorial gives a perspective on this process.

A highlight in the development of the discipline of cardiology was the establishment of the South African Heart Association (SAHA) in 1957. This was the first professional medical association to be formed in South Africa. The founding members were Dr Maurice Nellen and Prof Val Shrire (University of Cape Town), Prof JB Barlow and Prof Leo Schamroth (Wits University) and Prof Andries Brink (University of Stellenbosch). Nellen, a practising cardiologist in Cape Town, drafted the first constitution and devised an emblem for the SAHA.

This raised the value of the discipline of cardiology nationally and SAHA became a member of the World Federal Association of Cardiology. Cardiologists could henceforth participate in international cardiology conventions and arrange local annual conferences with the participation of invited overseas speakers.

SAHA is now the recognised voice of cardiology and can make proposals to the Health Professions Council. This body responds to requests and motivations from associations but does not initiate investigation into a society’s needs.

The South African Medical and Dental Council (SAMDC), as the Health Professions Council was previously known, set up a register for medical practitioners in 1928. This included recognised specialties such as internal medicine, surgery, gynaecology and obstetrics. The registration of a specialist cardiologist was motivated by the SAHA to the SAMDC. I was a member of the SAMDC, nominated by the Committee of University Principles (CUP) for the universities at that time. I was therefore able to guide this proposal successfully through the Council.

Cardiology specialists were required to have a basic higher qualification and training period (usually four years) as an internist, followed by two years in an approved cardiology facility. Training and updating of knowledge in South Africa has been done in close association with other countries. In the early years, England, Scotland, Ireland, Netherlands, Belgium and Germany were frequent training grounds for our postgraduate students. Latterly, the USA has increasingly become the place to acquire higher technological skills in cardiology.

Over the last 50 years, full training and higher qualifications have been available in South Africa. However, to keep abreast of the latest technology, close association is maintained with American, European and English colleagues.

Development of technology in South Africa has followed on the heels of international trends; we have not lagged behind. The 12-lead ECG remains the standard and first line of investigation. Echocardiography has emerged as an important tool for diagnosis. Imaging in many forms is now available to us: radionuclide perfusion MR, ECHO, CAT, SPECT and PET scanning and there can be little doubt that is the way forward. Non-invasive and invasive cardiology is rapidly replacing surgical procedures.

## National Heart and Stroke Foundation

The need was realised early on for a national heart foundation to promote cardiac health and educate the public. Over more than 20 years, representatives of SAHA met frequently in Johannesburg and Cape Town to discuss the establishment of such a foundation. Regional competitiveness, however, thwarted attempts to arrive at a consensus on the location. In the interim, each province established its own provincial heart foundation.

Dr Cyril Wyndham had focused attention on the high mortality rate from coronary artery disease, particularly among the Afrikaner population who were predisposed to familial hypercholesterolaemia (FH). In the commemorative articles appearing in this issue of the Journal, tribute is made to CH Wyndham and others who contributed to our knowledge in the field of cardiology.

The awareness of FH had stimulated public concern and the Medical Research Council (MRC) initiated a two-year campaign aimed at founding a national heart foundation. A national heart effort (NHE) was established to gain the support of the public and sponsors and to phase out the existing provincial heart foundations and convert them to a national heart foundation.

High-profile patrons were invited, and the prime minister, Mr BJ Vorster accepted as chief patron. The four provincial administrators and notable businessmen such as Anton Rupert and Punch Barlow also accepted patronship. A magnificent launch was held in Johannesburg at the Carlton Hotel with Sir Earnest Oppenheimer, Dr Anton Rupert and Punch Barlow among the many prominent citizens. The MRC produced a film on the topic of heart disease and held public meetings around the country, creating awareness of the risk factors underlying coronary artery disease and the need to contain and prevent the condition. The MRC personnel made huge personal financial contributions.

Consensus was obtained for the headquarters of the National Heart Foundation (NHF) of South Africa to be situated in Cape Town. The first meeting of the NHF as an independent organisation was convened at the MRC head office in 1982 with Mr Bill Davidson as chairman.

In 2007 the Stroke Society requested to join the NHF. The organisation, now known as the National Heart and Stroke Foundation, is a significant national asset.

## Heart research in South Africa

Early fundamental research in cardiology was done by a South African, WH Craib, with the support of a scholarship at the Johns Hopkins Hospital in Baltimore, USA. This is reflected in the tribute to Craib by Naidoo (page 8). Electrocardiography in South Africa was greatly enhanced by Leo Schamroth, as set out by Scott Millar (page 28).

Observations on unique or unusual aspects of heart disease have been a major stimulus for determining research direction in South Africa. These include: idiopathic dilated cardiomyopathy, (page 11), familial hypercholesterolemia and coronary artery disease (CAD) (page 18), and genetics and heart disease (page 57).

The consequences of CAD, such as angina pectoris, myocardial infarction and ischaemic heart disease are relatively rare events in the black population of South Africa. In whites on the other hand, CAD and its consequences have exploded in epidemic fashion over the last 50 years. Closer attention must be given to this situation. Is it possible that the black population could escape the devastation of this scourge of the western world?

Much original South African research came from individuals’ personal interest in and enthusiasm for research. A unique interest in the mitral valve and its diverse impacts on heart function and rhythm disturbances are set out on page 24, and studies on myocardial function and metabolism are commented on on page 37. The earth-shattering news of the first heart transplant in 1967 and consequent developments are retold on page 31.

Advances in the vascular arena of cardiology came from RH Goetz, who spent 20 years in South Africa doing outstanding research in the Department of Surgery at the University of Cape Town. He developed the Plethysmograph in 1934. Allan McLeod Cormack, a previous South African, was co-researcher and Nobel Prize winner in 1979 for his work on computerised axial tomography (CAT scan), which is of great value in imaging body organs and has application in cardiology.

Chronic rheumatic heart disease of known aetiology and pathogenesis is still rampant in South Africa, with its many impoverished communities. An active campaign to eradicate rheumatic heart disease is being waged under the direction of Bongani Mayosi of the University of Cape Town.

There are some less common heart conditions that may be unique to the black population, such as subvalvular mitral aneurysm. Other conditions have so far not been reported in blacks, such as the long QT syndrome and its variants. This may well be due to insufficient awareness, missed diagnosis or misdiagnosis. The condition, as seen in white South Africans, has its origins in European immigrants to South Africa.

## Development of research institutions in SA

No funding was available for research in the early years; researchers had to find their own means. However, the South African Institute of Medical Research (SAIMR) was established in 1914 by the directors of the gold mines who needed medical information on the effects of heat and dust on mineworkers. It also supported research in other areas that were relevant to heart disease, such as that of ARP Walker on nutrition and heart disease and A Shor on infection as a cause for CAD.

The number of medical schools has grown from only one in the country in 1922 to eight in 2008. They increasingly provide space and financial support for research.

State funding first came into being through the Council for Scientific and Industrial Research (CSIR), which was established in 1946. A committee for research in medical sciences (CRMS) to serve medical research was created for this purpose.

In 1969 the MRC was established and doctors could make a career of research. The MRC developed institutes and national research programmes, and promoted partnership with universities in the form of support for projects, and centres of excellence.

Laboratory research was well served with experimental animals supplied by a primate colony on the Karl Bremer Hospital site and an animal house in Delft. The Cape Province’s Department of Health was supportive in financing these facilities.

Foreign grants have increasing become a significant source of funding since 1994. Many programmes in cardiovascular disease have come into being since then.

## New organisations

PACE: Prevention of Arrhythmic Cardiac Events is an organisation established through the efforts of Suzanne Luscombe and Paul Brink to educate the public and create an awareness of conditions in South Africa that frequently lead to sudden, unexpected death in young people. The organisation provides support to affected patients and families.

## Die Afrikaanse vaktaal

Die Afrikaans Taal in Geneeskunde is ten volle ontwikkel as ’n dringende behoefte om geneeskunde in al sy fassette van pasiente kommunikasie, dienslewering, onderrig- en gevorderde navorsing te voldoen. Handboeke in kardiologie is opgestel.

## Publishing needs in cardiology

The publishing of research in cardiology has progressed with the founding of the *Cardiovascular Journal of Southern Africa* (CVJSA) in 1989. It subsequently changed its name to the *Cardiovascular Journal of South Africa* and is now incorporated in the *Cardiovascular Journal of Africa*. (CVJA). This has become a prestigious publication that is an internationally accredited, peer-reviewed journal, and has developed over a 20-year period. It is indexed and abstracted in SCI Expanded (SciSearch) and Journal Citation Reports/Science Edition. Both written and electronic versions are produced and an open access for full-length articles is available on PubMed.

The CVJA has become the official journal for the Pan-African Society of Cardiology (PASCAR), with regional editors in Kenya, Cameroon, Morocco, Nigeria and South Africa. A second journal, the *South African Journal of Diabetes & Vascular Disease* is now published in association with its British parent in England.

## CME out-reach programme

Under the auspices of the CVJA, out-reach programmes in cardiology have been developed. Areas (200-km) with a central village or town are identified and the local practising doctors are invited to attend. A teaching team of four or five doctors from academic departments and a private cardiologist are recruited. The meetings are held from Friday to Sunday, and sites such as Oudtshoorn, Tzaneen, Vryburg, Uitenhage, Ovambo, Rustenburg, Graaf-Reinet and Golden Gate have been targeted.

## Conclusion

Cardiology has become a leading discipline of South African medicine. It provides sophisticated services for clinical conditions at all levels and has the support of skilled cardiac surgeons. Training of under- and postgraduate students is outstanding and research has contributed greatly to our knowledge. The time may well have come for the SAHA to set up a physical ‘heart house’. This should be established at a neutral site and not be attached to any major institution.

